# Procalcitonin Identifies Bacterial Coinfections in Vietnamese Children with Severe Respiratory Syncytial Virus Pneumonia

**DOI:** 10.1155/2020/7915158

**Published:** 2020-05-09

**Authors:** Quyet Do, Tuan Minh Dao, Tran Ngoc Thi Nguyen, Quynh Anh Tran, Hau Thi Nguyen, Tam Thi Ngo

**Affiliations:** ^1^Respiratory Department, Military Medical Academy, Hanoi, Vietnam; ^2^Respiratory Department, National Children's Hospital, Hanoi, Vietnam; ^3^Surgical Department, National Children's Hospital, Hanoi, Vietnam; ^4^Faculty of Health Sciences, Thang Long University, Hanoi, Vietnam

## Abstract

This study assessed the diagnostic value of interleukin- (IL-) 6, high-sensitivity C-reactive protein (hs-CRP), and procalcitonin (PCT) in differentiating severe pneumonia caused by respiratory syncytial virus (RSV) alone and RSV with bacterial coinfections among Vietnamese children under 5 years old. A cross-sectional study on 70 children with severe RSV pneumonia was conducted. IL-6, hs-CRP, and PCT tests were performed. Receiver operating characteristic (ROC) analysis was employed to measure the diagnostic values of PCT, IL-6, and hs-CRP. Of 70 children, 11 children were confirmed to have bacterial coinfections. The most common bacterial coinfection was *Haemophilus influenzae*. This study underlined that inflammatory biomarkers such as PCT had a moderate-to-high capability of disseminating severe pneumonia children with RSV alone or RSV and bacterial coinfections. This may support clinicians in administrating appropriate antibiotics to children suffering from severe RSV pneumonia.

## 1. Introduction

Respiratory syncytial virus (RSV) is well recorded as a leading cause of lower respiratory tract infection (LRTI) in infants under 1 year old. RSV is annually attributable to 34 million LRTI episodes, accounting for 80% of LRTI cases globally [1, 2]. Moreover, this virus causes approximately 200,000 deaths among children aged less than five years, which mostly happened in resource-constrained countries [1].

Evidently, bacterial coinfections could elevate the severity of pneumonia in children compared to viral infections alone [3–5]. However, regular laboratory tests and radiographic results have poor ability to differentiate viral pneumonia patients with or without bacterial coinfections [6–8]. Thus, identifying biology markers to early and effectively discriminate these two circumstances is necessary for deciding whether antibiotics should be used or not. To date, some inflammatory biomarkers such as interleukin- (IL-) 6, high-sensitivity C-reactive protein (hs-CRP), and more recently, procalcitonin (PCT) are widely mentioned as potential mediators for diagnosing inflammatory illnesses [9, 10, 20]. PCT has been used in several guidelines to manage antibiotic use in children with respiratory diseases [11, 12] because a high level of serum PCT concentration frequently occurs in those with bacterial or parasitic infections [13].

This study is aimed at assessing the diagnostic value of hs-CRP, PCT, and IL-6 in differentiating severe pneumonia caused by RSV alone and RSV with bacterial coinfections among Vietnamese children under 5 years old.

## 2. Materials and Methods

### 2.1. Study Designs

We performed a cross-sectional study in 70 children who were confirmedly diagnosed with severe RSV pneumonia treated at the National Hospital of Paediatrics from January 2015 to March 2017 according to WHO 2013 standards [14]. Severe cases of pneumonia were defined when having a cough or difficulty in breathing plus at least one of the following main symptoms: (1) cyanosis or SpO_2_ < 90%, (2) severe respiratory distress (moaning and intercostal muscle external retraction), (3) could not drink or give up or vomit everything, coma or not awake, or convulsions. We excluded children who (1) were under 1 month of age and older than 5 years, (2) had non-RSV pneumonia (for example, pneumonia due to other viruses, pneumonia after drowning, chemical pneumonia, and aspiration pneumonia), (3) had chronic, associated congenital diseases (for example, airway malformation, congenital lung disease, liver failure, or kidney failure), (4) were coinfected with other viruses, and (5) were eligible to participate in the study but the parents or guardian did not agree to participate. The protocol of this study was approved by the institutional review board of Vietnam Military Medical University (Code 92/QD-HVQY).

### 2.2. Measurement

All patients after hospitalization were carefully examined for clinical symptoms by pediatrics. Their parents or guardians were also asked to collect information about demographic characteristics and history of illness. Two milliliters of blood samples was collected and centrifugated for biochemical tests. hs-CRP quantification was determined by turbidity measurement using Olympus AU 2700 machine. PCT quantification was measured by the luminescent immunization method, running on ADVIA Centaur of Siemens. Specimens were put into tubes without anticoagulants or with Li-Heparin and K3-EDTA anticoagulants. After collecting blood samples, they were centrifugated to extract serum or plasma. Meanwhile, IL-6 was quantified with the Bio-Plex Protein Array System of Bio-Rad.

Bacterial testing was performed by the Vitek 2 machine. The colorimetric method was used to identify the chemical biological properties of bacteria by changing the color of environmental wells. Moreover, an antibiogram method was applied using MIC (minimum inhibitory concentration) in order to measure turbidity which can monitor the development of microorganisms in the environmental wells. These two methods were performed according to the principle of light intensity reduction: the optical system used visible light to directly monitor the growth of microorganisms through the measurement of the intensity of the blocked light (or attenuation of light intensity) when light passes through a well. The system used 660 nm, 568 nm, and 428 nm wavelengths.

### 2.3. Statistical Analysis

Clinical and laboratory characteristics were compared between severe pneumonia children with RSV alone and RSV with bacterial coinfections by using Student's *t*-test (for age, respiratory rate, pulse rate, body temperature, and SpO_2_), Mann–Whitney test (white blood cells, lymphocyte, procalcitonin, high-sensitivity C-reactive protein, PaO_2_, and interleukin-6), and Chi-squared test (for gender, clinical features, and deaths after hospitalization). Receiver operating characteristic (ROC) analysis was employed to measure the diagnostic values of PCT, IL-6, and hs-CRP. This analysis informed some indices to measure the diagnostic accuracy including area under the ROC curve, sensitivity, specificity, positive predictive value (PPV), negative predictive value (NPV), positive likelihood ratio (LR+), negative likelihood ratio (LR-), and accuracy rate. Youden index was calculated to identify the optimal cut-off point. STATA software 12.0 was used to analyze data. A two-tailed *p* value of less than 0.05 was considered statistically significant.

## 3. Results

**Figure 1:**
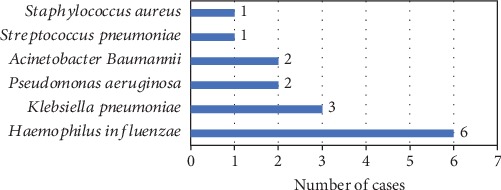
Distribution of bacterial pathogens in RSV pneumonia children with bacterial coinfections.

Of 70 children diagnosed with severe RSV pneumonia during the study period, 11 children were confirmed to have bacterial coinfections (15.7%). The most common bacterial coinfection was *Haemophilus influenzae* (6 cases), followed by *Klebsiella pneumoniae* (3 cases) and *Pseudomonas aeruginosa* (2 cases) (Figure 1).

The mean age of patients was 5.8 (SD = 8.2) months; 51.4% were males. There were two children that died after hospitalization (2.9%). The median of PCT was 0.3 (IQR = 0.12-1.2) ng/ml, the median of hs-CRP was 1.8 (IQR = 0.66-6.2) mg/dl, and the median of white blood cells was 9.7 (IQR = 7.05-12.6) g/dl.  Table 1 shows the demographic, clinical, and laboratory characteristics of RSV pneumonia patients. Patients with coinfections had a significantly higher proportion of fever, higher body temperature, PCT, hs-CRP, and a higher proportion of deaths after hospitalization compared to those with RSV alone (*p* < 0.05).

**Figure 2:**
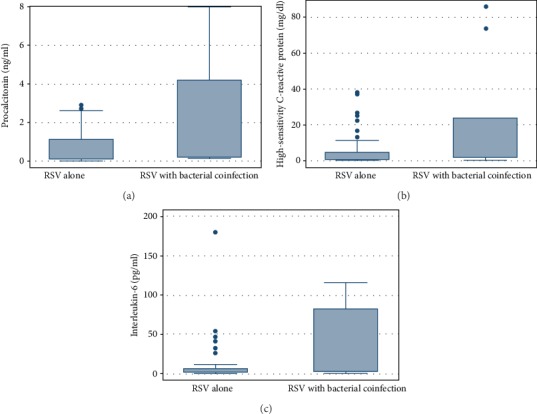
Box plot of (a) procalcitonin (PCT), (b) high-sensitivity C-reactive protein (hs-CRP), and (c) interleukin-6 (IL-6) levels on initial hospital visit between RSV pneumonia alone and RSV pneumonia with bacterial coinfections.

Figure 2 illustrates the distribution of PCT, hs-CRP, and IL-6 between severe pneumonia children with RSV alone and RSV combined with bacterial coinfections.

**Figure 3:**
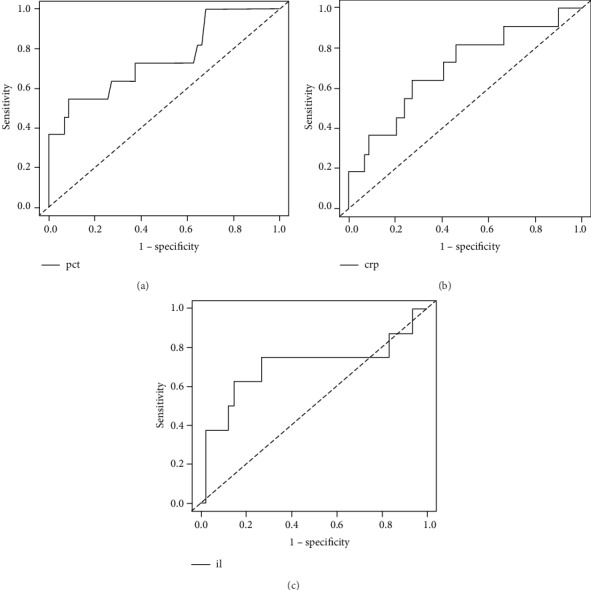
Receiver operating characteristics curve for discriminating between RSV pneumonia alone and RSV pneumonia with coinfections for (a) procalcitonin (PCT), (b) high-sensitivity C-reactive protein (hs-CRP), and (c) interleukin-6 (IL-6) on the initial hospital visit.

Results of receiver operating characteristics (ROC) curve analysis are shown in Figure 3. Overall, the area under curve was 0.749 (95%confidence interval = 0.571–0.927, *p* = 0.006) for PCT, 0.701 (95%confidence interval = 0.521-0.882, *p* = 0.03) for hs-CRP, and 0.703 (95% confidence interval = 0.439-0.968, *p* = 0.13) for IL-6. The differences among areas under the ROC curve for these three biomarkers were insignificant (*p* > 0.05).

Diagnostic values for PCT, hs-CRP, and IL-6 are presented in Table 2. Based on the Youden index, the optimal cut-off point for PCT was >2.25 ng/ml (sensitivity 55%, specificity 92%, positive predictive value 55%, and negative predictive value 92%). The optimal cut-off point for hs-CRP was >4.5 mg/dl (sensitivity 64%, specificity 73%, positive predictive value 30%, and negative predictive value 92%). Finally, the optimal cut-off point for IL-6 was >10.68 pg/ml (sensitivity 63%, specificity 85%, positive predictive value 42%, and negative predictive value 93%). The most accurate was for PCT with 83.6%, followed by IL-6 with 71.2%.

## 4. Discussion

Identifying biomarkers for the rapid detection of bacterial coinfections in RSV pneumonia among children hospitalized is essential in emergency circumstances. Our study highlights the potential role of serum PCT concentration in detecting and discriminating children with severe RSV pneumonia alone and severe RSV pneumonia with bacterial coinfections.

In this study, the optimal cut-off point for PCT was 2.25 ng/ml, which might be different from prior studies using PCT to discriminate these two types of patients in respiratory diseases. A study of Ahn et al. showed that PCT> 1.5 ng/ml had sensitivity 56% and specificity 84% to detect pneumonia patients having mixed bacterial coinfection or not [15]. Other studies by Chirouze et al. in patients with acute fever [16], Ingram et al. in H1N1 influenza patients [17], and Chua and Lee in severe acute respiratory syndrome [18] indicated the cut-off point for PCT was 0.4 ng/ml, 0.8 ng/ml, and 1.0 ng/ml, respectively. These variances might be attributable to the presence of different viruses and bacteria in each study. Moreover, our study employed children who suffered from severe RSV pneumonia, who actually had a high level of PCT compared to samples in other studies who had a variety of levels of disease severity.

In literature, serum PCT, hs-CRP, and IL-6 concentration have been used widely to differentiate patients suffering viral respiratory diseases alone and those with bacterial coinfections [9, 10, 20]. Most of the studies found that PCT was dominant in differentiating viral and bacterial infections compared to hs-CRP and IL-6 [9, 10, 19, 20]. Our finding in this study was in line with these previous works when we found that the accuracy rate of PCT was higher than that of IL-6 and hs-CRP. In addition, clinical symptoms and other laboratory test measures were not sufficient for diagnosing bacterial coinfections, which aligned with previous findings [6–8]. Serum PCT concentration above 2.25 ng/ml could detect bacterial coinfections more effectively, which can be applied in emergency cases. However, Laham et al. [20] and Baer et al. [21] found that some infants with pneumonia did not detect bacterial coinfections even when they had the highest value of PCT. This result was similar to another study in severe acute respiratory syndrome caused by SARS-CoV-2 virus [22] which suggested that antibiotic use might not be necessary for patients with high PCT. Therefore, in order to use antibiotic appropriately, further tests to confirm the existence of bacterial coinfection should be performed. Nonetheless, although PCT alone cannot be used to decide which antibiotics should be utilized, this is a vital marker that clinicians should require when making a decision rather than based on radiographic or blood cell count findings. Therefore, developing a rapid bedside PCT test is crucial in managing children experiencing severe RSV pneumonia.

This study contained several limitations. First, our sample size was small and conveniently recruited, which thus might reduce our generalizability. Second, our cross-sectional design has its own limitations due to its nature. Several recent studies argued that making decisions related to antibiotic use should use data of serum PCT concentration overtime via a longitudinal cohort rather than using the initial PCT level [23, 24]. In Vietnam, the PCT test has been covered in the health insurance scheme, which facilitates the use of PCT in routine monitoring and controlling bacteria in children with severe RSV pneumonia.

## 5. Conclusions

This study underlined that inflammatory biomarkers such as PCT had a moderate-to-high capability to disseminate severe pneumonia children with RSV alone or RSV and bacterial coinfections. This may support clinicians in administrating appropriate antibiotics to children suffering from severe RSV pneumonia.

## Figures and Tables

**Table 1:** Patients' characteristics between patients with RSV pneumonia alone and RSV pneumonia with bacterial coinfections.

	RSV alone (*n* = 59)	RSV with bacterial coinfections (*n* = 11)	*p* value
Age (months) (mean ± SD)	5.6 ± 8.5	6.8 ± 6.5	0.67
Male, *n* (%)	29 (49.2)	7 (63.6)	0.38
*Clinical features*			
Fever	22 (37.3)	9 (81.8)	<0.01
Rapid pulse rate	32 (54.2)	6 (54.6)	0.99
Runny nose	16 (27.1)	3 (27.3)	0.99
Wheezing	53 (89.8)	9 (81.8)	0.44
Diarrhea	14 (23.7)	2 (18.2)	0.69
*Vital signs (mean* ± *SD*)			
Respiratory rate (/min)	54.5 ± 7.2	54.1 ± 9.3	0.87
Pulse rate (/min)	153.3 ± 18.5	154.6 ± 21.5	0.83
Body temperature (°C)	37.3 ± 0.8	38.6 ± 1.0	<0.01
SpO_2_	88.5 ± 4.6	88.2 ± 4.0	0.82
*Initial laboratory findings (median, IQR)*			
WBC (×10^3^/mm^3^)	9.5 (7.1-12.1)	11.2 (5.4-17.3)	0.62
Lymphocyte (%)	4.6 (2.9-6.3)	3.4 (2.2-9.0)	0.46
Procalcitonin (ng/ml)	0.3 (0.1-1.1)	2.3 (0.2-4.2)	<0.01
High-sensitivity C-reactive protein (mg/dl)	1.5 (0.6-4.9)	5.7 (1.7-23.6)	0.04
PaO_2_	69.0 (50.0-82.0)	64.9 (54.0-92.0)	0.99
Interleukin-6 (pg/ml)	2.0 (1.5-6.1)	12.4 (3.0-82.8)	0.07
Deaths after hospitalization, *n* (%)	0 (0.0)	2 (18.2)	<0.01

SD: standard deviation; SpO_2_: oxygen saturation; WBC: white blood cell.

**Table 2:** Accuracy of diagnostic parameters regarding different cut-off points.

	PCT > 0.5 ng/ml	PCT > 2.25 ng/ml	IL − 6 > 10.68 pg/ml	IL − 6 > 100 pg/ml	hs − CRP > 4.5 mg/dl
Sensitivity	0.73 (0.39-0.94)	0.55 (0.23-0.83)	0.63 (0.25-0.92)	0.25 (0.03-0.65)	0.64 (0.31-0.89)
Specificity	0.63 (0.49-0.75)	0.92 (0.81-0.97)	0.85 (0.72-0.94)	0.98 (0.89-1.00)	0.73 (0.60-0.84)
Positive predictive value (PPV)	0.28 (0.17-0.68)	0.55 (0.33-0.83)	0.42 (0.15-0.72)	0.67 (0.26-0.92)	0.30 (0.19-0.67)
Negative predictive value (NPV)	0.93 (0.75-0.96)	0.92 (0.73-0.97)	0.93 (0.81-0.99)	0.89 (0.44-1.00)	0.92 (0.73-0.95)
Positive likelihood ratio (LR+)	1.95 (1.19-3.19)	6.44 (2.38-17.45)	4.29 (1.80-10.20)	12.00 (1.23-117.41)	2.35 (1.27-4.33)
Negative likelihood ratio (LR-)	0.44 (0.16-1.16)	0.50 (0.26-0.95)	0.44 (0.18-1.08)	0.77 (0.51-1.15)	0.50 (0.23-1.11)
Accuracy	64.3%	84.3%	82.1%	87.5%	70.0%

PCT: procalcitonin; hs-CRP: high-sensitivity C-reactive protein; IL-6: interleukin-6.

## Data Availability

The data used to support the findings of this study are available from the corresponding author. Requests for access to individual subject data may be made to Tran Ngoc Thi Nguyen, email dr.ngoctran259@yahoo.com.vn
